# Supplementary material for: Triiron Tetrairon Phosphate (Fe7(PO4)6) Nanomaterials Enhanced Flavonoid Accumulation in Tomato Fruits
Source: Nanomaterials (Basel). 2022 Apr 13;12(8):1341. doi: 10.3390/nano12081341 (PMC9028851; doi:10.3390/nano12081341)
Supplement: Supplementary file 1 [file nanomaterials-12-01341-s001.zip › nanomaterials-1631808-supplementary.pdf]

Supporting Information

Triiron Tetrairon Phosphate ($Fe_7(PO_4)_6$) Nanomaterials Enhanced Flavonoid Accumulation in Tomato Fruits

Zhenyu Wang [1,2], Xiehui Le [1,2], Xuesong Cao [1,2], Chuanxi Wang [1,2], Feiran Chen [1,2], Jing Wang [1,2], Yan Feng [1,2], Le Yue [1,2,*] and Baoshan Xing [3]

[1] School of Environment and Civil Engineering, Institute of Environmental Processes and Pollution Control, Jiangnan University, Wuxi 214122, China; wang0628@jiangnan.edu.cn (Z.W.); 6191403013@stu.jiangnan.edu.cn (X.L.); caoxuesong@jiangnan.edu.cn (X.C.); wangcx2018@jiangnan.edu.cn (C.W.); chenfeiran@jiangnan.edu.cn (F.C.); wangjing03282022@163.com (J.W.); 15251870663@163.com (Y.F.)

[2] Jiangsu Engineering Laboratory for Biomass Energy and Carbon Reduction Technology, Wuxi 214122, China

[3] Stockbridge School of Agriculture, University of Massachusetts, Amherst, MA 01003, USA; bx@umass.edu

* Correspondence: leyue@jiangnan.edu.cn; Tel.: +86-0510-85911911

Text S1. Ultra-performance liquid chromatography coupled with electrospray ionization and mass spectrometry (UPLC-MS/MS) conditions for auxin (IAA) and ABA detection.

UPLC condition details:

Briefly, 10 μL of extraction was injected in Vanquish UPLC (Thermo Fisher, Germering, Germany) with a T3 column (Waters ACQUITY HSS, 2.1 × 100 mm, 1.8 μm pore size) coupled to a Mass Q EXTATIVE (Thermal Fisher, Bremen, Germany). The mobile phases were altered as: A, 0.01 % formic acid (in water, v/v); B, 0.01 % formic acid (in ACN, v/v). The elution gradient was described below: 0 min, 5 % B; 1.5 min, 5 % B; 9 min, 70 % B; 10 min, 70 % B; 10.1 min, 5 % B; 15 min, 5 % B at the flow rate of 0.35 mL min^{-1}.

MS condition details:

PRM (Parallel Reaction Monitoring) was used as the collection mode of MS/MS and resolution of MS^2 was set as 17500. The isolation window and collision energy were 3.0 m/z and nce: 10, respectively. The negative polarity was selected as ESI mode and molecular ion ($[M-H]^-$) of IAA and ABA were determined as 174.0560 and 263.1289.

Text S2. UPLC-MS/MS conditions for naringenin, quercetin and rutin detection.

Sample preparation and extraction

The method of flavonoid extraction was modified from Martin-Rivilla et al. (2020) [82]. 100 mg of fruit powder was added to 1.5 mL of methanol/water (80:20, v: v). The mixture was vortexed for 1 min, sonicated for 5 min in ice bath, and centrifuged at 12000 rpm for 10 min at 4 °C to remove the sediment. Supernatants were collected for the UPLC-MS/MS analysis.

UPLC condition details:

Briefly, 5 μL of flavonoid extraction was injected in UPLC with a T3 column coupled to Mass Q EXTATIVE. The mobile phases were set as follow: A, 0.01 % formic acid (in water, v/v); B, 0.01 % formic acid (in ACN, v/v). The elution gradient was described below: 0 min, 5 % B; 1 min, 5 % B; 11 min, 95 % B; 12 min, 95 % B; 12.1 min, 5 % B; 14 min, 5 % B at the flow rate of 0.35 mL min^{-1}.

MS condition details:

PRM was used as the collection mode. The resolution of MS^2 was set as 17500. The isolation window and collision energy were 3.0 m/z and nce: 15, 30 and 45, respectively. Naringenin and quercetin were detected in positive mode and rutin was in negative mode.

Text S3. UPLC-MS/MS conditions for metabolomics.

UPLC condition details

In brief, 5 μL of extraction was injected in UPLC with a T3 column coupled to a Mass Q EXTATIVE. The binary mobile phases were consisted of A (water + 0.1 % formic acid) and B (acetonitrile + 0.1 % formic acid) and linear elution gradient was as follow: 0 min, 5 % B; 1.5 min, 5 % B; 10 min, 100 % B; 11 min, 100 % B; 11.5 min, 5 % B; 14 min, 5 % B at constant flow at 0.35 mL min^{-1}.

MS condition details

Nitrogen was used as sheath gas (35 L/min) and aux gas (15 L/min). The spray voltage was set as 3 kV (-3 kV for negative mode and 3 kV for positive mode) and capillary temperature was 320 ℃. The resolution of full MS was set as 70000 and scan range was acquired between 70 to 1050 m/z in full MS-dd MS^2 mode. The following dd-MS^2 product ion spectra were collected at resolution of 17500, isolation window of 1.5 m/z, and collision energy of nce: 20, 40, 60.

Text S4. Metabolic data processing.

The quality control (QC) samples were prepared by mixture of equal volume from each sample. And QC samples were inserted in sample sequences every 6 test samples. The raw files were analyzed by Compound Discoverer (CD) 3.1 performed on databases included Mass List, MzVault, Mzcloud and ChemSpider. The procedure of metabolite profiling process was conducted with peak detection (including filtration and noise elimination), peak matching, retention time alignment, peak annotation and compound identification on CD software [83]. The metabolites were discarded that RSD in QC samples were more than 30%. After combination of metabolites from both negative and positive mode, the selected metabolites were annotated by ID from HMDB or KEGG database. Based on metabolites abundance, principal component analysis (PCA), heatmaps and enrichment analysis were processed through Metaboanalyst 5.0 (https://www.metaboanalyst.ca/). For the selection of biomarkers from the metabolic analysis, VIP > 1 and $p < 0.05$ were set as standard.

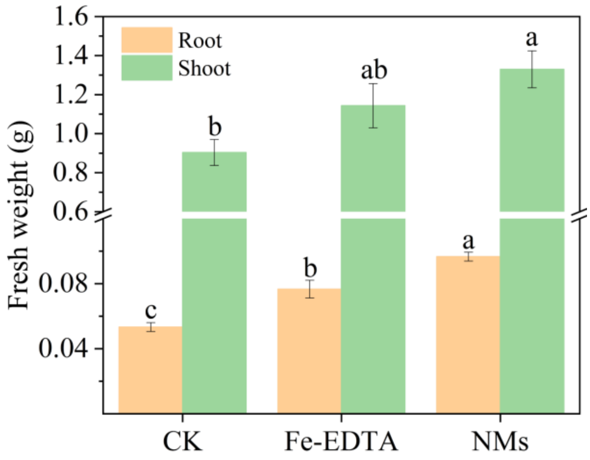

Figure S1 Biomass of roots and shoots in different treatments. Different letters above columns (a, b and c) represent significance of $p < 0.05$ and there is no significant difference between “ab” and “a”, nor between “ab” and “b”.

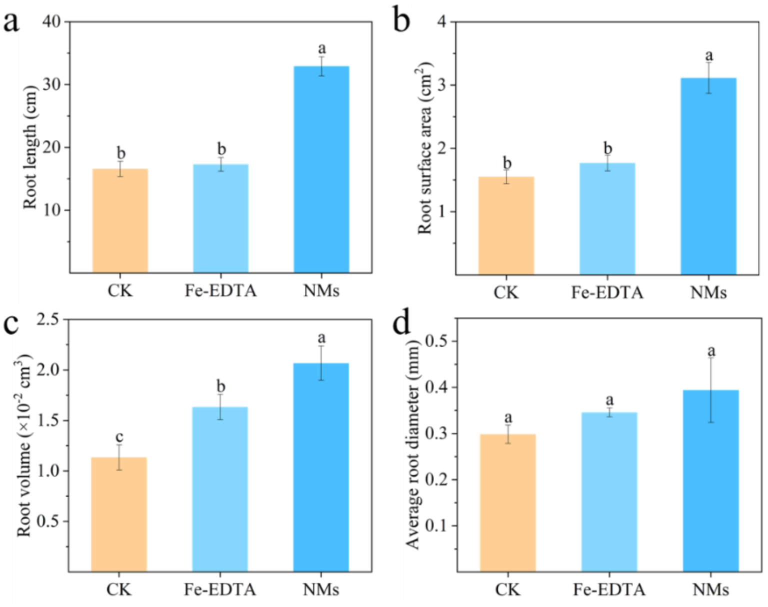

Figure S2 Root length (a), surface area (b), volume (c), and average diameter (d) of tomato seedlings exposed to $Fe_7(PO_4)_6$ NMs. Different letters above columns (a, b and c) represent significance of $p < 0.05$.

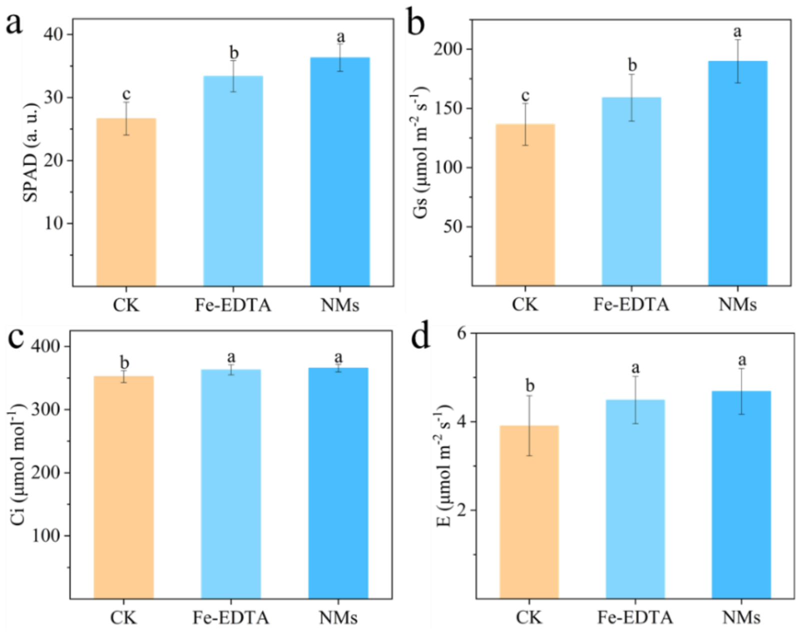

Figure S3 Photosynthetic parameters of tomato seedlings. (a) chlorophyll SPAD value; (b–d) Gs, Ci, and E value. Different letters above columns (a, b and c) represent significance of $p < 0.05$.

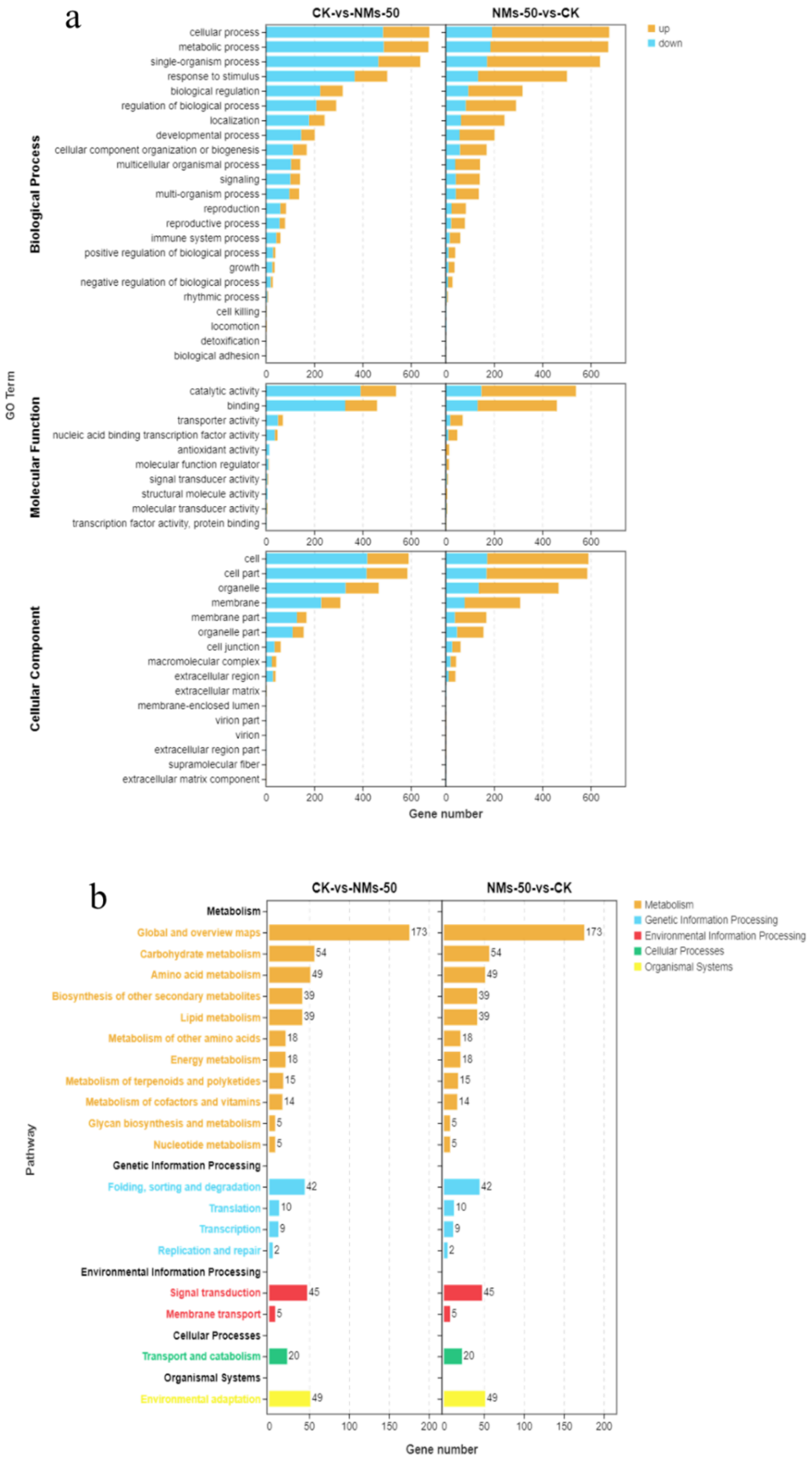

Figure S4 GO enrichment analysis of DEGs (a) and KEGG enrichment analysis (b) in tomato fruits.

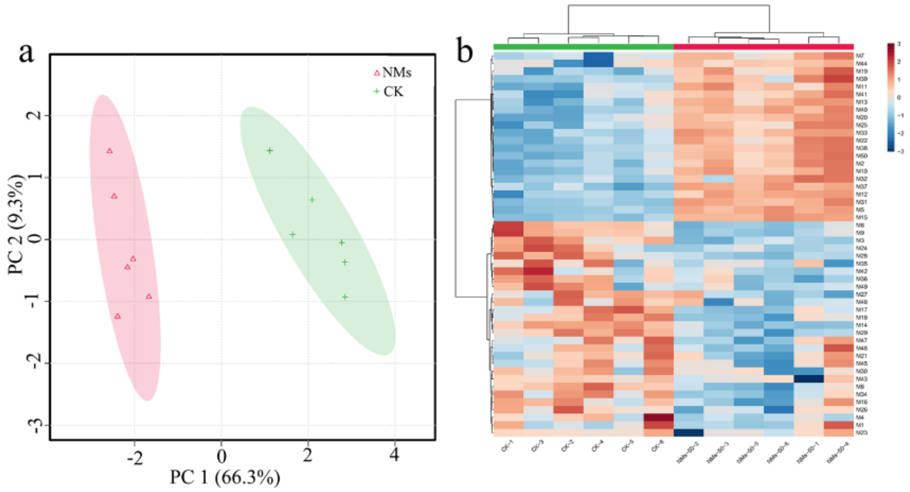

Figure S5 PCA plot (a) and DEMs heatmap (b) in tomato fruits. VIP value > 1 and $p < 0.05$ were set as DEMs standard.

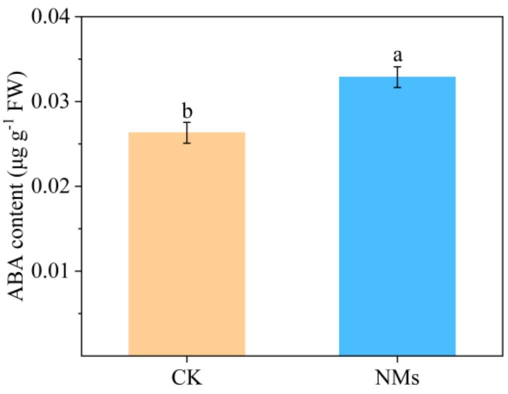

Figure S6 ABA content in fruits. Different letters above columns (a and b) represent significance of $p < 0.05$.

Table S1. The properties of soil used in this research

pH	Total organic carbon (g kg^{-1})	Total nitrogen (g kg^{-1})	Total phosphorus (g kg^{-1})	Total potassium (g kg^{-1})
5.9	97.0	27.5	0.3	7.7

Table S2. Primer sequences of flavonoids synthesis genes, PM H^+ ATPase genes and sucrose transporter genes.

Gene	Primer sets
SlPAL [84]	F: 5'-CGGTGAGGAGATTGATAAGG-3'<br>R: 5'-CATTTAGCAGATTGGAATAGGA-3'
SlC4H [84]	F: 5'-CTAGCTAACAACCCCGCCCA-3'<br>R: 5'-AACTCCTCCTGCCAACACCG-3'
Sl4CL [84]	F: 5'-GCTCTCTGCCCCGTAACCAA-3'<br>R: 5'-CCACGATGAAAAGCTCATCATCAT-3'
SlCHI [84]	F: 5'-ACAGGGGCAGAACTAACTGAAAA-3'<br>R: 5'-TCAAAAACTTGTCAACTGGAGCA-3'
SlCHS1 [84]	F: 5'-AGGGGTCGAAAGACCTTTAT-3'<br>R: 5'-TCCCTAGAGGTTGAAATGCT-3'
SlF3H [84]	F: 5'-TGGCGATCACGGTCATTATTTG-3'<br>R: 5'-GCATCTGGTGCTGGATTCTGG-3'
SlF3′H [84]	F: 5'-CTGGTGGCAAGAAAGGTGG-3'<br>R: 5'-CGAATGGGGTATGAAAAGTAAGTC-3'
SlFLS [85]	F:<br>5'-GCGCCGTCTCGCTCGGGAGGCGGCCGCGGAGTCTGAAAG<br>AGTTCTTATTTTG-3'<br>R:<br>5'-GCGCCGTCTCGCTCACATTTTTTCTTCTAACATGCAAAAA<br>ATTTTG-3'
Sl3GT [73]	F: 5'-CGAACGACGAAACACTGTTGA-3'<br>R: 5'-TGCAGCATAGATGGCATTGG-3'
SlCAC [84]	F: 5'-TGGGTGTGCCTTTCTGAATG-3'<br>R: 5'-GCTAAGAACGATGGACCTAATG-3'
LHA1 [86]	F:5'-GGATTGCTTTCACCCG-3'<br>R:5'-TTCGCTCCCATCACGACACC-3'
LHA2 [86]	F:5'-TGAAATCGCTAGGCAACG-3'<br>R:5'-ATTCTATCACCCACTCACC-3'
LHA4 [86]	F:5'-AGGGAATGGCAGGCTCT-3'<br>R:5'-GGGTGGTGCTTCAAAA-3'
Tubulin [86]	F:5'-GTGTTACTTGCTGTTTGAGA-3'<br>R:5'-TTTGTGCTCATCTTACCC-3'
SlSUT1 [19]	F:5'-GAACTCCCGGAGAAAGAAGAGCTAGA-3'<br>R:5'-TCGCATCACCGACTTGTCCACC-3'
SlSUT2 [19]	F:5'-TCTGAAGCAGCAGGAAGTGGA-3'<br>R:5'-CGGTGGAGGAAGAGGTAGATTAG-3'
SlSUT4 [19]	F:5'-CACCATCAACTGTGCCAATCTAAA-3'<br>R:5'-CAACACCTGGGAAATACTTGAAAAT-3'
SlTIP4 [19]	F:5'-ATGGAGTTTTTGAGTCTTCTGC-3'<br>R:5'-GCTGCGTTTCTGGCTTAGG-3'

Table S3. Hydrodynamic diameter and zeta potential of $Fe_7(PO_4)_6$ NMs.

Hydrodynamic Diameter (D_h)	716.89±60.32 nm
Zeta potential	-14.37±1.09 mV

Table S4. Fpkm value of DEGs in pathways from sucrose metabolism to flavonoid synthesis pathway.

Genes	Treatment		Genes	Treatment	
	CK	NMs		CK	NMs
SlSS	165.48±30.34	333.32±18.89	*SlEPSPS-1*	45.22±1.10	38.15±1.29
SlTPS6	18.60±0.97	43.00±1.50	*SlCM2*	28.47±0.81	18.51±1.87
SlTPP	42.59±4.35	93.14±1.55	*Sl4CL1*	89.04±2.25	107.51±1.76
SlTKL-1	12.59±1.85	16.64±0.28	*SlCHI*	0.17±0.02	0.38±0.09
SlDHQ-SOR	33.24±0.43	28.18±1.37	*SlMYB12*	11.45±0.96	16.06±1.55
SlPYL4	39.87±1.07	115.01±9.74			

Table S5. Differentially expressed metabolites (DEMs) selected of tomato fruits in CK and upon $Fe_7(PO_4)_6$ NM exposure by VIP > 1 and $p < 0.05$. Name of metabolites and its codes were corresponding to heatmap (Figure. S5b).

Code	Name	Code	Name
M1	L-Glutamyl-L-glutamic acid	M26	1-Methyladenine
M2	Trehalose	M27	9-HpODE
M3	Ferulic acid	M28	Shogaol
M4	Ethyl sorbate	M29	Sedanolide
M5	Ethyl myristate	M30	Salicylic acid
M6	D-Serine	M31	Rutin
M7	D-Mannose 6-phosphate	M32	Quercetin
M8	DL-Stachydrine	M33	Ribono-1,4-lactone
M9	DL-Carnitine	M34	Phenethylamine
M10	Uridine 5'-diphosphogalactose	M35	Palmitoleic acid
M11	D-Cysteine	M36	N-Feruloyloctopamine
M12	D-(+)-Camphor	M37	Naringenin
M13	Cysteinylglycine	M38	N-Acetylputrescine
M14	Corchorifatty acid F	M39	N8-Acetylspermidine
M15	Citral	M40	N6-Acetyl-L-lysine
M16	Betaine	M41	Methylmalonic acid
M17	Arachidonic acid methyl ester	M42	L-Valine
M18	6-Pentyl-2H-pyran-2-one	M43	L-Tyrosine
M19	5'-S-Methyl-5'-thioadenosine	M44	L-Serine
M20	5-Hydroxyindole	M45	L-Pyroglutamic acid
M21	4-Oxoproline	M46	Linoleoyl ethanolamide
M22	4-Guanidinobutyric acid	M47	L-(+)-Citrulline
M23	3-Hydroxypicolinic acid	M48	Imidazoleacetic acid
M24	Tomatidine	M49	Hexadecanedioic acid
M25	1-Methyladenosine	M50	Glycylproline

Table S6. Relative content of metabolites detected in sucrose metabolism pathway.

Name	CK	NMs
UDP-glucose	$(3.20±0.26)×10^5$	$(3.76±0.54)×10^5$
Trehalose	$(2.29±0.11)×10^5$	$(2.94±0.09)×10^5$
D-Glucose-6P	$(1.58±0.10)×10^6$	$(1.74±0.14)×10^6$
